# Statewide Outbreak of *Neisseria meningitidis* Serogroup Y, Sequence Type 1466 — Virginia, 2022–2024

**DOI:** 10.15585/mmwr.mm7343a3

**Published:** 2024-10-31

**Authors:** Meredith Robinson, Jenny Crain, Brittany Kendall, Victoria Alexander, Elena Diskin, Dawn Saady, Corryn Hicks, Angela Myrick-West, Paige Bordwine, Denise Sockwell, Emily Craig, Amy Rubis, Lucy McNamara, Shalabh Sharma, Rebecca Howie, Daya Marasini, Henju Marjuki, Ana Colón

**Affiliations:** ^1^Virginia Department of Health; ^2^Division of Consolidated Laboratory Services, Virginia Department of General Services; ^3^National Center for Immunization and Respiratory Diseases, CDC.

**Summary**

**What is already known about this topic?**

Meningococcal disease is a serious illness; U.S. outbreaks are uncommon. Vaccination of a defined population at risk (e.g., college students or persons experiencing homelessness) is recommended during outbreaks.

**What is added by this report?**

In a Virginia outbreak, 36 cases of serogroup Y meningococcal disease occurred during August 2022–March 2024; seven (19.4%) patients died. Most patients were aged 30–60 years, an age group not generally at increased risk for meningococcal disease. Patients lacked common exposures or affiliations. Vaccination was recommended for close contacts within the patient age range.

**What are the implications for public health practice?**

Occurrence of meningococcal disease outbreaks in populations without well-defined risk groups might prompt exploration of novel control strategies, such as selective vaccination of close contacts.

## Abstract

Invasive meningococcal disease (IMD) is a severe illness that can have devastating effects; outbreaks are uncommon in the United States. Vaccination is the preferred control measure for IMD outbreaks when a defined population at risk (e.g., college students or persons experiencing homelessness) can be identified. In August 2022, the Virginia Department of Health (VDH) began investigating an IMD outbreak in Virginia’s Eastern Health Planning Region, prompted by the detection of four confirmed cases within 8 weeks. Clinical isolates available from three cases were characterized as *Neisseria meningitidis* serogroup Y, sequence type 1466. A subsequent statewide investigation identified 36 genetically related cases, including seven deaths (case fatality rate = 19.4%) as of March 1, 2024. A majority of patients (63.9%) were in an age group (30–60 years) not generally considered at increased risk for IMD; 78.0% were non-Hispanic Black or African American. No common exposures, affiliations, or risk factors were identified, and a defined population could not be identified for vaccination. VDH recommended quadrivalent (serogroups A, C, W, and Y) meningococcal conjugate vaccination of a subset of close contacts of patients based on IMD risk factors and age range similar to that of patients with identified cases. IMD outbreaks might affect populations without established IMD risk factors. Lack of a well-defined population at risk might prompt exploration of novel control strategies, such as selective vaccination of close contacts.

## Introduction

Invasive meningococcal disease (IMD), caused by the bacterium *Neisseria meningitidis*, is a serious illness that manifests primarily as meningitis or meningococcemia (a bloodstream infection) (*1*). The Advisory Committee on Immunization Practices (ACIP) recommends routine meningococcal vaccination for some persons based on age and disease risk (*2*). In an outbreak setting, CDC recommends offering vaccination to a defined target group considered to be at increased risk based on a common affiliation, geographic community, or shared characteristics (*3*). In August 2022, the Virginia Department of Health (VDH) began investigating an IMD outbreak in Virginia’s Eastern Health Planning Region.

## Investigation and Results

### Initial Cases

On August 12, 2022, VDH learned of two patients with IMD hospitalized in the Virginia Eastern Health Planning Region. *N. meningitidis* serogroup Y (NmY) was identified in blood specimens from both patients. Two additional NmY IMD cases were reported in the Eastern Region during June–July 2022; in contrast, during the preceding 10 years, the Eastern Region averaged only one IMD case per year, and Virginia averaged eight IMD cases per year statewide. No common exposures, epidemiologic linkages, or specific risk factors were identified among the four cases. All available *N. meningitidis* isolates were sent to CDC for antimicrobial susceptibility testing and whole genome sequencing (WGS). This activity was reviewed by CDC, deemed not research, and was conducted consistent with applicable federal law and CDC policy.*

### Case Finding and Reporting

On September 2, 2022, CDC notified VDH that the three available isolates from the four initial cases, and a fourth isolate from a fifth case identified on August 9, 2022, were the same sequence type (ST) 1466, within clonal complex (CC) 174 (*4*). With this confirmation of genetic relatedness among clinical isolates, outbreak-specific case definitions were established. A confirmed outbreak case was defined as identification of *N. meningitidis* (NmY ST1466 and within 33 single nucleotide polymorphisms of another outbreak case, or *N. meningitidis* untyped, and not known to be ciprofloxacin- or penicillin-resistant) via culture or polymerase chain reaction in a specimen from a normally sterile body site in a resident of or visitor to the Eastern Region with onset of IMD symptoms (fever, headache, nausea, vomiting, photophobia, or stiff neck) after June 1, 2022. A probable outbreak case was defined as one identified through epidemiologic linkage, in which *N. meningitidis* was not detected, but all other confirmed outbreak case definition criteria were met. Based on these case definitions, VDH conducted active case finding by monitoring syndromic surveillance^†^ and reviewing medical records of patients with suspected meningitis or meningococcemia.

During November 9, 2022–July 14, 2023, seven NmY ST1466 cases were identified in the Southwest Region (five) and Central Region (two) among patients without a history of travel to the Eastern Region. As a result, the regional criterion was removed from the outbreak case definition in August 2023.

### Case Characteristics

During June 12, 2022–March 1, 2024, a total of 36 confirmed and one probable IMD outbreak cases were identified (Figure) (Table). The probable case occurred in an unvaccinated health care provider who became symptomatic 5 days after providing direct care to a patient with a confirmed outbreak case. The health care provider received postexposure prophylaxis (PEP) before specimen collection and specimens were negative for *N. meningitidis* by polymerase chain reaction testing and culture. Confirmed outbreak cases were identified among four of VDH’s five health planning regions, including the Eastern Region (25), Southwest Region (six), Central Region (three), and Northern Region (two). The majority of patients (58%) primarily lived in urban areas.

**FIGURE:**
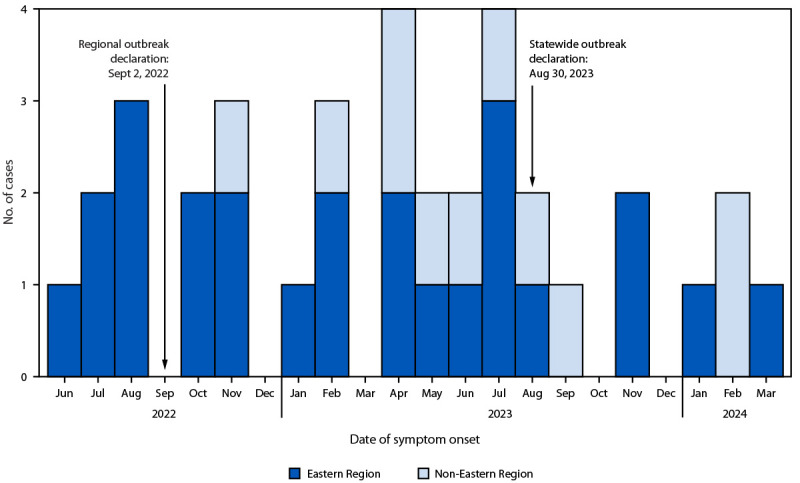
Invasive meningococcal disease associated with an outbreak of *Neisseria meningitidis* serogroup Y, sequence type 1466 (N = 36) — Virginia, 2022–2024

**TABLE:** Epidemiologic and clinical characteristics of laboratory-confirmed meningococcal disease cases (N = 36)* — Virginia, June 12, 2022–March 1, 2024

Characteristic	No. (%)
**Age group, yrs**	
Median (IQR)	47 (16–82)
0–29	4 (11.1)
30–59	23 (63.9)
≥60^†^	9 (25.0)
**Sex**	
Female	15 (41.7)
Male	21 (58.3)
**Race**	
Black or African American, non-Hispanic	28 (77.8)
White, non-Hispanic	8 (22.2)
**Meningococcal vaccination status**	
Unvaccinated	35 (97.2)
Received 1 dose^§^	1 (2.8)
Received 2 doses	0 (—)
**Outcome**	
Survived	29 (80.6)
Died	7 (19.4)
**Infection type**	
Meningococcemia	24 (66.7)
Meningitis	3 (8.3)
Septic arthritis	2 (5.6)
Meningococcemia and septic arthritis	2 (5.6)
Pericarditis	1 (2.8)
Meningococcemia and meningitis	1 (2.8)
Meningococcemia and myocarditis	1 (2.8)
Meningococcemia and pneumonia	1 (2.8)
Meningitis and pneumonia	1 (2.8)
**Immunosuppression status**	
HIV-positive	5 (13.9)
Other immunosuppression^¶^	1 (2.8)
No medical record evidence of immunosuppression	30 (83.3)
**Diabetes**	
Yes	10 (27.8)
No or unknown	26 (72.2)
**History of, or current cigarette smoking**	
Yes	23 (63.9)
No or unknown	13 (36.1)
**History of, or current marijuana use**	
Yes	12 (33.3)
No or unknown	24 (66.7)
**History of, or current cocaine use**	
Yes	5 (13.9)
No or unknown	31 (86.1)
History of, or current injection drug use**	
Yes	2 (5.6)
No or unknown	34 (94.4)

* One contact who provided direct patient care met the probable case definition but was excluded from the overall outbreak case count.

^†^ Seven patients were aged ≥65 years and two were aged 60–64 years.

^§^ Patient not known to have HIV infection–acquired *Neisseria meningitidis* serogroup Y infection 16 years after receipt of 1 Menomune polysaccharide A/C/Y/W vaccine dose.

^¶^ Medical record documentation of immunosuppression because the patient was taking organ transplant antirejection medication.

** Self-reported or medical record evidence of use of heroin, methamphetamines, or both.

Overall, 28 (78%) of the 36 patients with confirmed cases were non-Hispanic Black or African American (Black) and 21 (58%) were male; the median patient age was 47 years (range = 16–82 years). A majority of cases (64%) occurred in persons aged 30−60 years. All patients required hospitalization. Seven patients died (case fatality rate [CFR] = 19.4%) from IMD complications. Among the fatal cases, the median patient age was 41 years (range = 33−56 years); three cases (43%) occurred in patients with one or more underlying health condition. Meningococcemia without other clinical syndromes was the most common clinical reason for seeking care, identified among 24 patients (Table). Clinical symptoms of meningococcemia included fever, nausea, vomiting, diarrhea, and muscle aches. Five patients had HIV infection, one with HIV treatment documented. One patient was receiving immunosuppressive medication after organ transplantation. Ten patients had diabetes. Thirty-five patients had no evidence of previous meningococcal vaccination against NmY (e.g., Menactra, MenQuadfi, or Menveo), including four patients aware of their HIV diagnosis for whom vaccination is routinely recommended. One patient had received 1 dose of meningococcal polysaccharide vaccine (Menomune-A/C/Y/W) 16 years before symptom onset.

Approximately two thirds of patients (23; 64%) were current or former tobacco smokers. Fourteen patients were current or former substance users. Five patients cited employment in the construction industry; however, no common employer or work site was identified. No common affiliations, exposures, or risk factors other than shared demographic characteristics were identified. The average social vulnerability index score^§^ of patients’ residential U.S. Census Bureau tracts was 0.63 (range = 0.05–1) overall and 0.73 for fatal cases, compared with 0.4 for all Virginia residents (*5*).

### Isolate Characteristics

Isolates from 34 confirmed cases were available for WGS; all were characterized as NmY: ST1466/CC174 with a recent common ancestor sharing a fine-typing^¶^ genetic profile: PorA (P1.21,16), FetA (F3–7), and PorB (3–35) (*4*). In contrast to some recently identified ciprofloxacin- and penicillin-resistant NmY strains (*6*), genotypic resistance markers were absent, and ciprofloxacin and penicillin sensitivity was inferred. Before this outbreak, IMD cases caused by NmY ST1466 were uncommon in the United States.

## Public Health Response

VDH declared a community outbreak of NmY in the Eastern Region on September 2, 2022, and disseminated a clinician letter on September 23, encouraging health care providers to maintain a high index of suspicion for IMD and to ensure that eligible patients were up to date with routine quadrivalent (serogroups A, C, W, and Y) meningococcal conjugate (MenACWY) vaccination (*2*). VDH also sent a letter to HIV health care providers emphasizing the increased risk for IMD among persons with HIV infection and that MenACWY vaccine is recommended for persons aged ≥2 years with HIV infection (*2*). On March 6, 2023, VDH issued a press release and launched an outbreak response website** to raise public awareness. A statewide outbreak was declared on August 30, 2023, via a news release, and additional communications were distributed to health care providers and the public. Virginia residents were advised to ensure that they were up to date with recommended meningococcal vaccinations and to not delay seeking care if they experienced IMD symptoms. In consultation with CDC, VDH evaluated options for vaccination of a defined population (*3*,*7*,*8*); however, no common affiliation or shared characteristics could be identified among cases that would guide delineation of a feasible population for vaccination.

### Management of Close Contacts

VDH conducted standard public health interviews with patients to identify close contacts. A close contact was defined as a person who experienced lengthy contact^††^ with a person with a confirmed case, or exposure to the patient’s oral secretions, within 10 days of the patient’s symptom onset. Per established recommendations, VDH emphasized the need for timely receipt of PEP, consisting of a short course of a recommended antibiotic (e.g., rifampin, ciprofloxacin, or ceftriaxone), for all close contacts (*3*). One probable case was identified in a health care contact; no additional cases occurred among identified close contacts.

### Vaccination Strategy

In December 2022, VDH issued a recommendation to local health departments in the Eastern Region to offer 1 dose of MenACWY vaccine, in addition to antimicrobial PEP, to a subset of close contacts of persons with IMD (*3*). Vaccination was recommended for close contacts if 1) they had established risk factors for IMD (e.g., HIV infection) and were not up to date with routine MenACWY vaccination as recommended by ACIP (*2*), or 2) they were aged 30–60 years and had not received a dose of MenACWY vaccine within the preceding 5 years. This recommendation was expanded statewide in August 2023 and revised to include all close contacts aged ≥11 years because additional cases were identified among patients outside the 30–60-year age range. No additional cases were reported among identified close contacts following this recommendation. Limited data are available on MenACWY vaccine administration during this outbreak; however, based on anecdotal reports from local health department staff members, vaccine acceptance was low among all indicated groups.

## Discussion

Several features of this ongoing outbreak are unusual for NmY disease in the United States. A majority of patients (86%), including six patients with fatal cases, did not have typical meningitis symptoms. CFR (19.4%) was higher than the national average CFR for NmY during 2017–2022 (6.5%–17.2%), although NmY-associated CFRs ≤27% were reported during 2015–2016.^§§^ The relatively high CFR in this outbreak might represent random variation, or reflect a hypervirulent strain or delays in diagnosis and treatment due to atypical disease symptoms. Further, residence of patients in areas of higher social vulnerability might have been associated with challenges in accessing medical care, increasing the likelihood of poor outcomes. In addition, although NmY commonly affects adults aged ≥65 years, in this outbreak, the median patient age was 47 years, and approximately three quarters of patients were aged <65 years, although a substantial proportion had underlying conditions that might have increased their risk for IMD.

Although common exposures and affiliations were not identified, this outbreak disproportionately affected Black persons. In the Southwest Region, for example, 83% of outbreak patients were Black compared with 12% of the region’s residents (*9*). This finding might reflect carriage of NmY ST1466 among contacts of a shared social network. In addition, the disproportionate impact of NmY ST1466 on Virginia’s HIV-positive population is consistent with reports of a national increase in IMD among persons with HIV infection (*10*). In 2016, ACIP recommended routine administration of MenACWY vaccine for persons with HIV infection (*2*). Previous reports have identified low rates of MenACWY vaccination among U.S. patients with a new diagnosis of HIV infection (*10*), highlighting a need for strategies that might improve vaccine acceptance, such as provider education.

Lack of a well-defined population at risk during this outbreak posed a challenge to implementing vaccination as an outbreak control strategy. Although antimicrobial chemoprophylaxis of close contacts of patients with meningococcal disease is important for preventing secondary cases, CDC does not routinely recommend vaccination of close contacts. In this outbreak, selective vaccination of close contacts (in addition to antimicrobial prophylaxis) was recommended in an effort to prevent additional cases among a population presumed to be at risk. Unfortunately, low vaccine acceptance precluded evaluation of the impact of this intervention on outbreak progression.

### Implications for Public Health Practice

A rapid public health response, guided by a comprehensive epidemiologic investigation, is necessary for controlling meningococcal disease outbreaks. Especially in outbreaks in which no common exposures, epidemiologic linkages, or specific risk factors among cases are identified, determination of genetic relatedness among isolates through WGS can be critical to guiding decisions about outbreak declaration and control strategies. The unique epidemiology of this outbreak demonstrates the potential for uncommon strains of *N. meningitidis* to spread in populations not previously considered at high risk for meningococcal infection. Lack of a well-defined outbreak group might prompt exploration of novel control strategies, such as selective vaccination of close contacts.

## Footnotes

* 45 C.F.R. part 46.102(l)(2), 21 C.F.R. part 56; 42 U.S.C. Sect. 241(d); 5 U.S.C. Sect. 552a; 44 U.S.C. Sect. 3501 et seq.

† Syndromic surveillance was conducted through the Virginia Electronic Surveillance System for the Early Notification of Community-based Epidemics. https://www.vdh.virginia.gov/surveillance-and-investigation/syndromic-surveillance/

§ Social vulnerability refers to the resilience of communities (the ability to survive and thrive) when confronted by external stresses on human health, stresses such as natural or human-caused disasters, or disease outbreaks. Socially vulnerable populations are especially at risk during public health emergencies because of factors such as socioeconomic status, household characteristics, racial and ethnic minority status, or housing type and transportation. The social vulnerability index is measured from 0–1; the higher the number, the more vulnerable the population.

¶ Fine typing refers to mapping of specific genes common to the same sequence type or clonal complex below the serogrouping level, facilitating verification that the isolates share the same genetic profile.

**
https://www.vdh.virginia.gov/surveillance-and-investigation/meningococcal-disease-outbreak-response

†† Persons in the same household, roommates, or anyone with direct contact with the patient’s oral secretions.

§§
https://www.cdc.gov/meningococcal/php/surveillance/index.html#cdc_generic_section_5-data-reporting
